# A Right-Sided Approach to Anterior Communicating Artery Aneurysms: A Case Review and Technical Report

**DOI:** 10.7759/cureus.45863

**Published:** 2023-09-24

**Authors:** James Brazdzionis, Imran Siddiqi, Tye Patchana, Maxwell A Marino, Daniel Welsh, Sanjay C Rao, Dan E Miulli

**Affiliations:** 1 Neurosurgery, Riverside University Health System Medical Center, Moreno Valley, USA; 2 Neurosurgery, Georgetown University, Washington, DC, USA; 3 Neurological Surgery, Kaiser Permanente Fontana Medical Center, Fontana, USA

**Keywords:** cerebral aneurysm surgery, unruptured cerebral aneurysm, ruptured cerebral aneurysm, anterior communicating artery aneurysm, right sided approach, microneurosurgery, aneurysm clip

## Abstract

Anterior communicating artery (ACoA) aneurysms are the most frequently encountered type of intracranial aneurysm. ACoA aneurysms may require treatment depending on clinical presentation, size, risk of rupture, and ruptured status. In patients where treatment is indicated, options entail endovascular securement or clipping. Under the clipping umbrella, surgical approaches traditionally entail a pterional craniotomy and its modifications such as the lateral supraorbital approach. Sidedness of this craniotomy has been a topic of debate. To discuss this we present a case and technical report with nuances of the approach wherein a 48-year-old female presented with the worst headache of her life. The patient was found to have a ruptured wide-necked 7.2 x 8.1 x 5.8 mm ACoA aneurysm more eccentric to the left and fed from the left A1 intertwined with a frontopolar branch, numerous perforators and the recurrent artery of Heubner. The patient underwent a successful clipping from a right-sided approach. As such, with appropriate skull base drilling, exposure, optimization of brain relaxation, and a generous opening of the Sylvian fissure bilateral internal carotid arteries, anterior cerebral arteries with both A1 and A2 segments, middle cerebral arteries, the ACoA, and the relevant anatomy can be appropriately visualized from a right-sided approach. Therefore, an approach is described to optimize exposure to allow for nearly all anterior communicating aneurysms to be clipped from a right-sided pterional approach.

## Introduction

Anterior communicating artery (ACoA) aneurysms are the most common type of intracranial aneurysm, accounting for 23-40% of ruptured aneurysms and 12-15% of unruptured aneurysms [1]. These aneurysms require prompt evaluation and may require treatment due to the risk of rupture if they are determined to be high-risk [2]. Surgical treatment of these aneurysms is challenging due to their complex anatomical structures and proximity to important blood vessels and structures. Microsurgical clipping and endovascular embolization are both viable treatment options with individualized pros and cons. Over the past few years, the advancement of neuro-interventional techniques and devices has led to an increase in the use of endovascular securement for both ruptured and unruptured ACoA aneurysms [3]. These endovascular techniques have benefits in regard to the potential for shortened hospital courses and a less invasive approach. However, microsurgical clipping still can offer several advantages in the treatment of unruptured ACoA aneurysms, including low aneurysm recurrence rates, and when performed correctly, low complication rates [4].

Surgical planning is critical to ensuring appropriate selection and reducing potential complications. It is necessary to determine the structure of the ACoA and perforating arteries, whether there is a fenestration deformity and the relationship between bilateral A1-A2 before surgery. The shape and size of the aneurysm itself and its location relative to adjacent blood vessels must also be considered. Various surgical approaches are available, including the pterional, interhemispheric, and subfrontal approaches, and their modifications and newer approaches such as the lateral supraorbital and supraorbital keyhole approaches. Among these, the pterional approach remains a common and effective surgical method for treating most ACoA aneurysms [5,6]. With a pterional approach, the question of sidedness for craniotomy has been a topic of debate. However, a right-sided approach from the non-dominant hemisphere can be utilized in nearly all cases of ACoA aneurysm clipping.

## Case presentation

**Figure 1:**
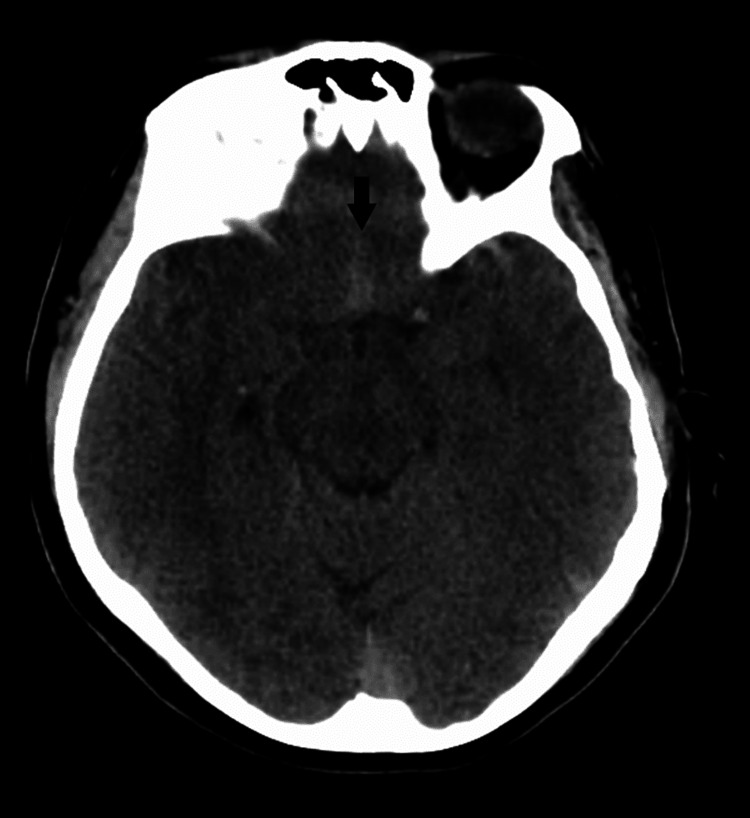
Pre-operative non-contrast CT head with identified subarachnoid hemorrhage in the interhemispheric fissure in the region of the anterior communicating artery. A pre-operative non-contrast CT head was obtained. An arrow points towards hemorrhage in the subarachnoid spaces and interhemispheric fissure in the region of the anterior communicating artery. CT: computed tomography

**Figure 2:**
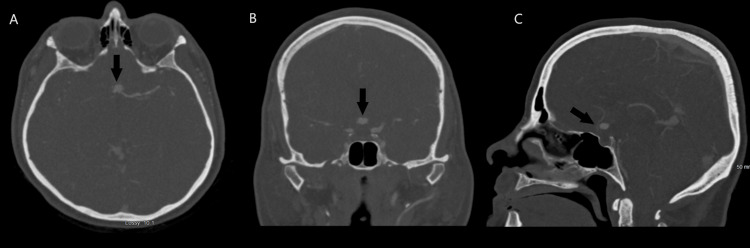
Pre-operative computed tomography angiogram of the head identifying a 7.2 x 8.1 x 5.8 mm anterior communicating artery aneurysm. Pre-operative computed tomography angiography was performed identifying the anterior communicating aneurysm. Axial (A), sagittal (B), and coronal views (C) are presented with arrows indicating the aneurysm.

**Figure 3:**
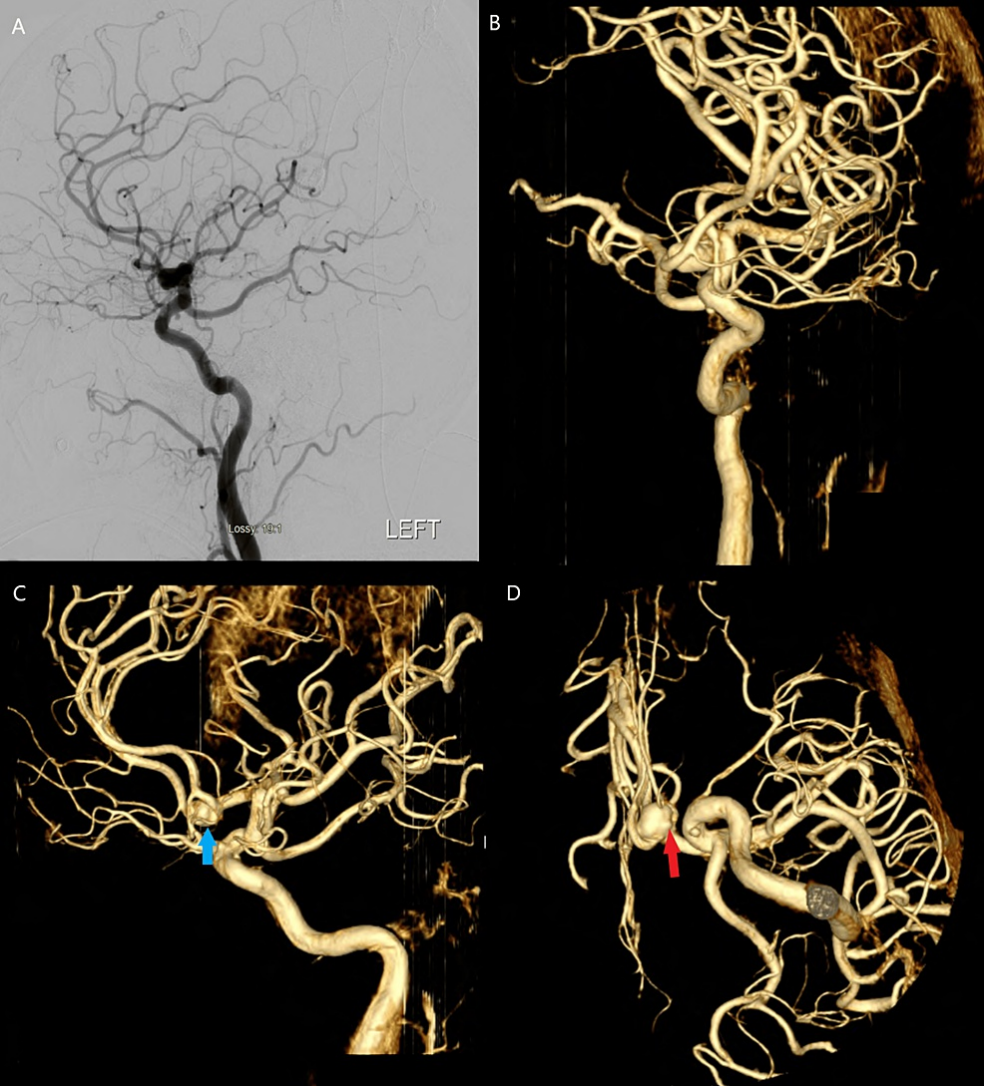
Pre-operative digital subtraction angiographic and three-dimensional reconstructions of the anterior communicating artery aneurysm Pre-operative digital subtraction angiographic views are seen in panel A, with three-dimensional reconstructions demonstrated in panels B, C, and D identifying the associated daughter blebs and intertwined perforators, recurrent artery of Heubner, and frontal polar branch. The daughter blebs are demonstrated in panel D with the red arrow and the intertwined vasculature is demonstrated in panel C with the blue arrow.

A 48-year-old female presented to the emergency department for a severe headache that occurred four days prior to admission while the patient was at work. She did not lose consciousness and proceeded to go home after work. After the headache did not improve for several days she sought care in the emergency department. The patient underwent a computed tomography (CT) scan and CT angiogram of the head and was found to have a subarachnoid hemorrhage focused on the ACoA (Figures 1, 2). The CT angiogram of the head identified a 7.2 x 8.1 x 5.8 mm ACoA aneurysm more eccentric to the left. The patient was admitted to the intensive care unit and remained neurologically intact aside from headache with a Hunt-Hess score of 1 and a modified Fisher grade of 1. The patient was taken for a diagnostic cerebral angiogram where the aneurysm was found to be wide-necked (4.5mm) with two blebs arising from the lateral and inferior of the dome. There were multiple vessels with origins at the base of the aneurysm including the recurrent artery of Heubner, an enpassage left frontal polar branch arising from the left A2 to the fundus of the aneurysm, a dominant left A1 feeding the bilateral A2. The diagnostic angiogram is seen in Figure 3. After a multidisciplinary discussion with neuro-interventional and neurosurgery and a review of the imaging, the patient underwent a right-sided craniotomy for clipping of her ACoA aneurysm.

A right-sided pterional craniotomy was selected for the approach as is routine for nearly all ACoA aneurysms at our institution. This approach was selected to avoid the left hemisphere and with appropriate dissection, skull base drilling, and brain relaxation, it is possible to appropriately evaluate the contralateral anterior cerebral artery, perforators, and relevant nearby structures to safely treat these aneurysms within the region. After initial incision and dissection paying careful attention to separate the superficial temporalis fascia and fat pad containing the frontal branch of the facial nerve, a craniotomy was planned to ensure the root of the zygoma, superior orbital rim, and zygomatic arch were visualized. A burr hole over the MacCarty keyhole was made and a craniotomy flap was turned. Using an extradural dissection, the sphenoid ridge was exposed, and a generous amount of sphenoid was drilled and removed with a Lempert to ensure optimal fissure dissection. Dissection was carried out to the meningo-orbital band and the dura was then separated from the bone until the extradural internal carotid artery (ICA) was visualized to ensure proximal control.

**Figure 4:**
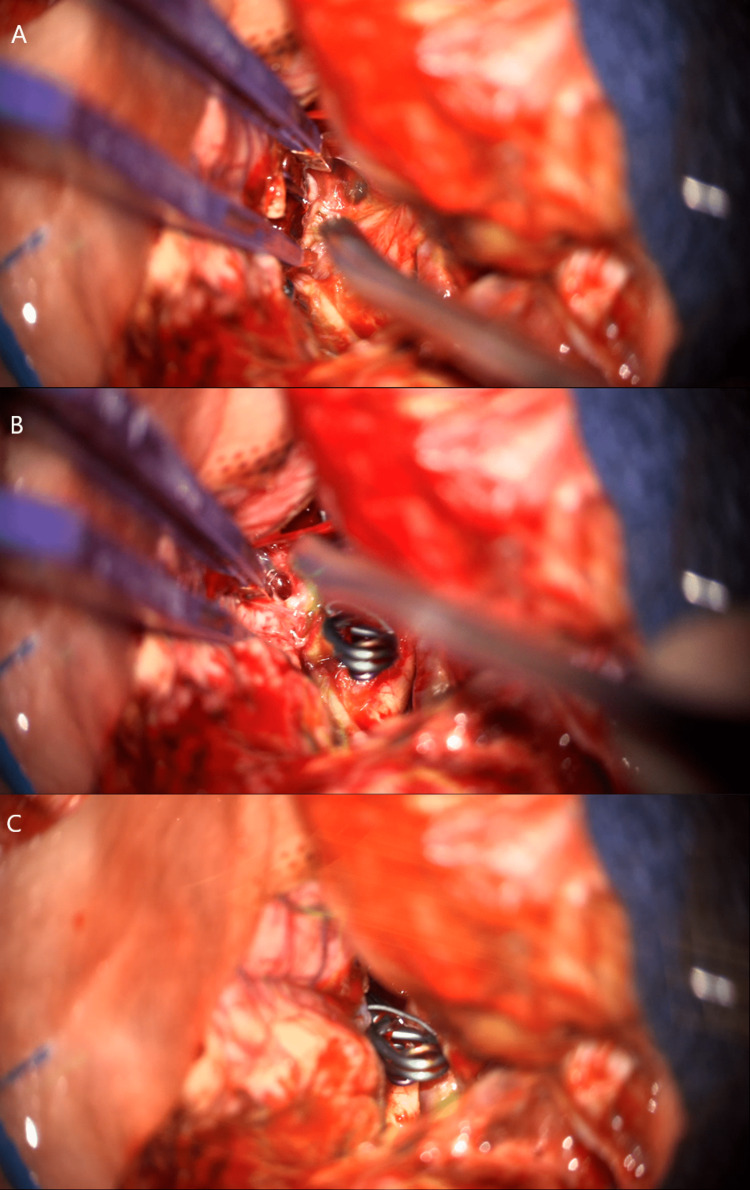
Intraoperative microscope images obtained post-clipping. It is noted that the clip construct is retracted towards the frontal lobe in panel A wherein contralateral structures and vessels are well visualized. Panel B identifies structures including perforators located at the dorsal surface of the aneurysm. Panel C identifies the clip construct and an example of the widely split Sylvian fissure allowing for optimal brain relaxation.

**Figure 5:**
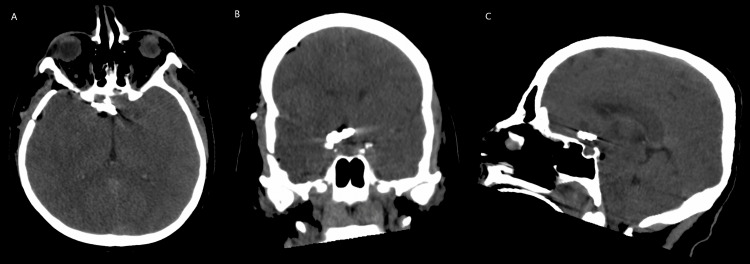
Post-operative non-contrast computed tomography of the head Axial (A), coronal (B), and sagittal (C) views were obtained identifying appropriate clipping of the anterior communicating artery aneurysm without new hemorrhage.

A linear incision was made under the microscope in the direction of the Sylvian fissure. After retracting the dura, the Sylvian fissure was opened generously using arachnoid dissection from a distal to proximal fashion to visualize proximal ICA, the origin of the posterior communicating artery, anterior cerebral artery (ACE) origins, and contralateral ICA. The dissection was carried out medially until the ACoA, ACoA aneurysm, ipsilateral, and contralateral A1 and A2 were visualized. At this time the base of the aneurysm was carefully inspected to ensure all en passage vessels were protected, and perforators and the recurrent artery of Heubner were identified. The contralateral dominant A1 noted on the angiogram was identified and a temporary clip was placed on it. Careful dissection was completed over the dome of the aneurysm. Using a tandem clip technique, four clips were placed on the ACoA aneurysm causing cessation of flow within the aneurysm and preserving the perforators and vessels. The temporary clip on the contralateral A1 was then removed and patent flow was confirmed with an indocyanine green angiography run and microdoppler. The craniotomy was then closed in a layered fashion. Screencaps of this approach and post-clipping evaluation are seen in Figure 4B. A postoperative CT was obtained (Figure 5).

Treatment of ACoA aneurysms ranges from observation for small low-risk aneurysms to surgical or endovascular therapies for those that are ruptured or at higher risk. In those that are selected to undergo operative intervention with clipping, multiple techniques have been utilized in the past. The mainstays of these approaches are the pterional approach and the interhemispheric approach. In the review of our practice, for which 27% of the ruptured and unruptured anterior communicating aneurysms seen in patients at our institution have undergone clipping, we have found that with generous dissection of the Sylvian fissure, optimization of brain relaxation and skull base dissection that nearly all ACoA aneurysms can be approached using a right-sided pterional craniotomy. In order to do so, nuances must be considered. Therefore we will describe an approach to clip the ACoA aneurysm from the right side.

## Discussion

Description of surgical procedure

Within the local practice, the preferred approach for most typical ACoA is a pterional approach over the non-dominant frontal lobe which is traditionally right-sided. This is unless there is the presence of a giant aneurysm with a superior trajectory or that fills from the contralateral A2 which can necessitate a contralateral or interhemispheric approach. A left-sided approach is traditionally utilized if there is a large left-sided clot requiring evacuation as this will simultaneously allow for appropriate removal to optimize the outcome and provide ease of access to the ACoA aneurysm. In certain cases, such as ruptured ACoA aneurysms that extend directly anterior and adhere to the optic chiasm, giant aneurysms, or cases of unsuccessful clipping of ACoA aneurysms with a pterional approach, or multiple aneurysms of the ACoA and pericallosal artery, the interhemispheric precallosal approach may be preferred. Although, for most lesions located within the subfrontal, cavernous sinus, subtemporal, and Sylvian fissure regions, the pterional approach is the preferred surgical method [4,5].

For a right-sided approach, initially, the patient is positioned slightly turned toward the left with a roll under the right shoulder. Ensuring the anesthesiologist has achieved appropriate sedation to reduce the risk of rapid blood pressure increases which may cause intraoperative or pre-operative rupture. The head is placed in Mayfield pins with the head rotated about 60 degrees to the left. This rotation is carefully computed to optimize rotation but not to create an iatrogenic occlusion or kink the left jugular vein to maximize appropriate cerebral venous outflow. The vertex of the head should be toward the floor with the zygoma as the highest point within the operative field. This positioning allows gravity to pull the frontal lobe away from the skull base. To improve the trajectory to the target of interest, maximal removal of the frontal, sphenoid, and temporal bone projections is required. The pterional approach is fundamental to most anterior cranial procedures and can be enhanced through several modifications, including extradural resection of the frontal bone, orbital roof ridges, and sphenoid bone ridges. Resecting the anterior clinoid, roof of the optic canal, orbitozygomatic osteotomy, addition of a temporal craniotomy, and addition of a petrosal approach is not usually needed but should be a familiar tool in the armamentarium of the seasoned neurosurgeon if necessary to increase exposure.

The skin incision and initial opening are done in the standard fashion, and with enough skin and muscle retraction to get to the roof the orbit to drill it flat and access to the inferior aspect of the temporal lobe. Next, the surgeon makes a single-entry burr hole at the MacCarty keyhole, and the bone flap is turned. The surgeon then removes the protrusions of the sphenoid ridge with a high-speed drill until the periorbital is visible at the lateral aspect of the superior orbital fissure. Extradural bone removal is a critical step for later achieving adequate intradural exposure. The temporal bone is removed with a rongeur to a level flush with the floor of the middle fossa.

Once extradural dissection is complete, the dura is opened. After the dural opening, the first step is to dissect the Sylvian fissure. This is completed from lateral to medial and if necessary, from medial to lateral. Once the dissection is complete, it's essential to open the fissure generously. If the choice is to dissect the fissure from lateral to medial, it is crucial for the surgeon to confirm the correct opening in the arachnoid layer to prevent damage to blood vessels, especially the M3 and M4 branches. In situations where the brain is severely swollen, an initial medial dissection may not be possible. To alleviate swelling, an external ventricular drain (EVD) may be necessary. If edema is anticipated, an EVD may be placed before entering the surgical suite, especially in ruptured cases. In non-elective cases, the EVD is traditionally initially set at 20 mmHg and intracranial pressure should be transduced. This is to reduce the risk of rapidly decreasing transmural pressure along the aneurysm for the potential risk of rupture. The EVD can then be gradually lowered over several hours to days, with the goal of setting it to 6mmHg or less by the time the patient goes into surgery. This approach helps to relax the brain more than usual, increasing the chances of adequately opening the Sylvian fissure for greater visualization and exposure of the H-complex. It is essential to adjust the EVD setting to a closer approximation of 6mmHg before opening the dura, as this decrease to 6mmHg may better approximate the change in transmural pressure anticipated upon dural opening; significant changes in pressure should be avoided to prevent aneurysm re-rupture. However, once the dura is open, a significant change in transmural pressure is typical due to the loss of this additional restrictive layer. If the patient has severe cerebral edema pre-operatively, the possibility of even performing open surgery for clipping may depend on the condition of the patient. Persistent collapsed ventricles and high intracranial pressures despite EVD placement and appropriate decompression with a depressed Glasgow Coma Scale (GCS) score of 8 or less and a high Hunt-Hess grade of four or five are indications that edema is maximal, and surgery may not be feasible.

In ruptured cases, upon identification of the carotid artery and oculomotor cistern, the surgeon should navigate medially and anticipate encountering additional hematoma. To ensure a clear pathway, it is recommended to dissect open the membranes and release cerebrospinal fluid (CSF) while aspirating blood. The appropriate dissection of the Sylvian fissure is important to visualize the optic nerve and carotid artery as well as the middle cerebral artery (MCA) and distal ACA, which can be identified by following the carotid artery distally and turning medially. An adequate amount of MCA visualization indicates that pressure is low enough and sufficient hematoma has been aspirated. The surgeon can then proceed along the ACA towards the ACoA while staying on the ventral side. It is not advisable to cross to the dorsal aspect of the A1 segment as this is where the neck or the dome is typically and can contain important perforators from the ACoA, A1, and A2 arteries. Once the A1 segment has been dissected anteriorly, the surgeon reaches the ACoA and must stay on the ventral side while identifying the recurrent artery of Huebner (RAH), which may branch off from either A1 or A2 until reaching the opposite A1. The surgeon can also identify the contralateral optic nerve and carotid artery and follow contralaterally to A2. During dissection, the surgeon must understand the relationships between the visualized dome, neck, and perforators for planning clip placement. Opening additional cisterns allows for the release of CSF for continued brain relaxation to increase exposure and facilitate clip placement.

In ruptured cases, the site of rupture, covered by adherent pia-arachnoid, brain, and clot in the interhemispheric fissure, should not be disturbed. The left A1 segment is identified for contralateral proximal vascular control, and sharp dissection is used to develop a path for clip placement at the junction formed by the aneurysm neck, ACoA, and right and left A2 segments. Temporary occlusion of both A1 arteries should not be done in a cavalier or typical fashion but may be necessary in unusual circumstances to reduce tension in large or complex aneurysms prior to final dissection and clipping, which can be done while minimizing risks using systemic hypertension and brain-protective anesthesia. If feasible, the contralateral A2 segment may be dissected, but in some cases, excessive bleeding may prevent the identification of the A2 segment. To further relax the brain, the lamina terminalis may be opened out of the third ventricle. This will allow for better visualization of the anterior aspect of the ACoA and the neck of the aneurysm. Once the base of the communicator is freed up, the contralateral A2 can usually be identified.

The neck and dome position of the aneurysm should be determined. At times, under very limited conditions, temporary straight clips may be placed on the A1 segment and, if further necessary, the contralateral A2. The neck of the aneurysm should be dissected to visualize both sides and sometimes the RAH must be dissected off before a clip is placed. The clip should be placed on the neck as low as possible. When selecting the appropriate clip shape, it is essential to consider the neck's orientation and its relation to nearby blood vessels. It is also crucial to determine the direction of the neck and dome and to visualize the aneurysm clip's shape from under the microscope. Additionally, one should consider the clip applier's characteristics, such as modulation or rotation, angulation, shape, or grips, as the appliers can be just as vital as the clip's shape in enabling the surgeon to position it correctly. With the ability to adjust the applier's angle, the surgeon may be able to secure the clip in the desired position. It is crucial to ensure that all blood vessels are off the aneurysm before carefully dissecting off any brain tissue from the dome in a proximal to distal fashion. After clip placement, securement should be evaluated and if unsatisfactory, a second clip may need to be placed above the first one, and the proximal clip should be replaced. It is important to verify that no blood vessels are retained within the clip and to use micro-Doppler and indocyanine green to assess good blood flow within nearby vessels and no flow in the aneurysm. The aneurysm and clip are carefully rotated to ensure that the medial hypothalamic perforators are free from the clip blades. To preserve the perforators, due to the variations in angle between ACoA perforators and the A2 ACA which varies between 30 and 180 degrees [7]. The mass effect from the clip itself needs to be considered as it has been associated with compressive optic neuropathy, of which re-exploration led to near-complete restoration of vision in the few patients described [8].

Laterally projecting aneurysms may have additional nuances, especially in those that additionally point posteriorly. In these cases, during dissection, it is important to make sure the surgeon can visualize around the distal neck. When placing clips, it may be beneficial to place a temporary clip higher on the neck first to ensure placement is safe and does not involve perforators or other vasculature prior to placing a permanent clip in a more appropriate position.

Superior projecting aneurysms can present technical difficulties for clipping as well. The dome in these cases may be adherent to one or both A2 arteries. A plane should be carefully dissected between the anterior aspect of both A2 and the posterior aneurysm fundus. Then the perforators adhered to the dome should be meticulously freed with microdissectors prior to clip placement [9].

Using a micro-Doppler and indocyanine green flow within the aneurysm and nearby vessels may be assessed. If there is no blood flow within the aneurysm, the surgeon can puncture the dome of the aneurysm if it has not already collapsed using a 31-gauge needle to provide decompression. If there is any bleeding from the dome, the aneurysm clip will need to be readjusted. Furthermore, opening the aneurysm’s dome will allow for appropriate decompression from any mass effect of a large aneurysm and will allow for better contralateral assessment. Once opened the surgeon can better see the neck contralaterally and should carefully evaluate the contralateral perforators.

Finally, a complete indocyanine green video angiography can be performed under the microscope to ensure that there is sufficient blood flow to the cerebral circulation and perforators while excluding the aneurysm. To prevent the avulsion of an aneurysm with a dome adherent to the chiasm, it is advisable to dissect on the opposite side of the blood vessel from where the aneurysm is, to avoid pulling it. The technique for clip placement varies depending on the approach taken. If possible, opening the interhemispheric fissure can provide more space for clip placement and manipulation. One can go above, over, and then behind the communicator to locate the neck of the aneurysm for clip placement. Alternatively, if the aneurysm is coming more anteriorly from the communicator, staying on top of the communicator is recommended. The plane containing the neck, which is inferior to the ACoA should be developed while being careful not to interfere with the RAH. After this, the opposite A1 can be accessed to remove any hematoma in ruptured cases, which helps to relax the brain. Finally, the lamina terminalis can be opened for additional drainage of CSF and improved brain relaxation. Closure is completed in a standard pterional craniotomy fashion, including dural closure with a continuous suture and placement of a dural retention suture in the center of the bone flap, securing the bone flap with a standard cranial fixation system.

Preoperative considerations

Computed tomography angiography (CTA) and formal diagnostic cerebral angiogram (DSA) are the preferred methods for pre-operative evaluation for most cerebral aneurysms. Several studies suggest that a 256-slice CT angiogram can provide beneficial information for aneurysm clipping on the aneurysm's origin, neck width, and trajectory angle [10]. Preoperatively, the A1 segment's length can be determined using angiography. However, when dealing with ACoA aneurysms, the resolution of the CTA may be affected by the bony skull base and clinoids, making it difficult to obtain detailed information about their morphology. In such cases, a formal DSA with 3D reconstruction sequences should be completed to better understand the complex vascular territory, particularly for small ACoA aneurysms that can resemble blister aneurysms. DSA has other additional benefits including a better understanding of the flow dynamics of the surrounding vessels and the aneurysm itself. Before surgery, it is important to examine several anatomical factors, including the size and projection of the aneurysm’s dome and neck, the side of A1 dominance, the height of the ACoA complex from the cranial base, as well as the orientation of the ACoA complex in both the sagittal and coronal planes.

Anatomical considerations

A thorough understanding of the anatomical characteristics of the aneurysm and a careful review of preoperative imaging results are crucial for the successful completion of surgery and the minimization of postoperative complications. The transition from the A1 to A2 segments of the ACA defines the origin of the ACoA, which is often associated with an unbalanced anatomy of A1s’ calibers resulting in an aneurysm dome that projects in the direction of flow through the dominant A1. When exposing the ACoA complex, up to 14 arteries may need to be identified, of which 10 are paired arteries: right and left A1s’ and A2s’, RAH, orbitofrontal arteries, and frontopolar arteries. The remaining four include the ACoA itself, the hypothalamic perforators, a third A2 segment, and a possible aberrant early-branching callosomarginal branch. Occlusion of the ipsilateral RAH can cause a caudate stroke and the classic triad of contralateral face and arm weakness and aphasia. In most patients, the recurrent artery’s origin lies within 4 mm of the ACoA, allowing for localization of the ACoA but also implies the surgeon must be careful not to disrupt or involve this critical vessel within their clip construct. The ACoA usually resides in an oblique plane, impacting the dissection strategy, and surgical anatomy variations can make dissection more complex. A thorough study of the preoperative angiogram is essential before clip application to prevent poor outcomes. The hypothalamic perforators from the ACoA must be visualized and preserved to minimize the risk of neuropsychological and memory deficits [11,12]. Visualization can be done from either approach side if there is sufficient exposure and brain relaxation.

Identification of these perforators is critical to optimizing outcomes in ACoA aneurysm clipping. Perforators of the A1 include medial lenticulostriate (MLA) and RAH arteries. In a retrospective study of 104 ruptured ACoA patients, 23.1% had a perforator infarction where the use of temporary clips and intraoperative rupture were significantly associated with infarction [13]. Infarction of these perforators can cause neurocognitive effects, endocrinopathy, and electrolyte dysfunction including hyponatremia and insufficient oral intake. Anatomically this is due to the cortical supply of the MLA and RAH which supplies the internal capsule, hypothalamus, olfactory region, anterior commissure, and fornix. Most perforators from A1 occur in the proximal portion of the A1 segment. Protection of these perforators can be done through careful preoperative localization, planning, and ensuring dissection occurs distal to proximal [14].

Variations of the RAH need to be considered and understood based on careful evaluation of intraoperative anatomy and pre-operative imaging. The RAH is the largest vessel of the MLAs [15]. Its origin varies in anatomical studies and is found to arise in order from most to least: A2 48%, ACoA 43%, doubled 6%, missing 5%, and A1 4%. Injury to this artery can result in contralateral facial and limb weakness, and if on the dominant hemisphere may result in aphasia [1].

ACoA aneurysms are distinct from other simple intracranial bifurcation aneurysms due to their complex origins [16]. It is essential to note that ACoA aneurysms typically originate from the A1-ACoA or A2-ACoA junction, rather than from the ACoA alone. Therefore, a generous view of the ipsilateral ACA segments is critical for optimal neck exposure and clip application. Since the medial walls of the A1 and A2 are in the operator's blind spot, the surgeon may underestimate the extension of the aneurysm neck on the ipsilateral A2, resulting in incomplete clipping, which is the most common cause of intraoperative premature rupture or residual aneurysm filling [12]. This issue can be mitigated by appropriate positioning to allow the frontal lobe to fall away, sufficient exposure, drilling of the orbit and sphenoid, and if necessary, placement of EVD.

Clinical considerations and nuances

In treating an ACoA aneurysm, it is important to evaluate the clinical condition of the patient and the reasoning for securement. In an elective clipping with a large but high-risk ACoA aneurysm, a traditional right-sided approach can be ideal. However, there are some nuances that must be considered. First, if able, it is good to know the dominance pattern of the individual. Although in our described approach there is minimal brain retraction due to optimization of brain relaxation, reasons for avoiding the dominant hemisphere should be considered. Likewise, if there is right-brain dominance, a left-sided approach may be appropriate. Surgeon-handedness may also play a role in decision-making. For most surgeons, a right-sided approach may be technically easier due to right-handedness, however, if they are left-hand dominant a left-sided approach may be technically easier due to the trajectory of the working corridors and surgical cone with the working left hand staying closer to the skull base and orbit and away from the cortical structures themselves.

An additional consideration includes specific decisions during ruptured cases. For patients that present with a ruptured ACoA aneurysm, there can be significant clot burden and edema that may limit the operative view. In these cases, it can sometimes be necessary to perform a gyrus rectus (GR) resection. Partial GR resection has been performed on ruptured ACoA aneurysms to improve exposure to the ACoA complex through the junctional triangle which is made up of the distal A1, proximal A2 ACA segments, and the medial surface of the GR. GR resection is especially useful in those with poor clinical grade, acute stage, superior, and posterior projecting, or high-positioned aneurysm. Quantitative studies in cadaveric heads show that GR resection increased exposure of ipsilateral A2 from 2 to 4 mm, contralateral A2 from 3 to 4 mm, and exposure to contralateral RAH and orbitofrontal arteries from 5 and 8 to all 10 specimens respectively. A comparative study of 194 patients wherein 52 patients received GR resection, showed that resection resulted in no apparent clinical effects on outcome. Overall, a GR resection can lead to a safer surgery through greater exposure of A2s and contralateral OF and RAH arteries in those for which it is necessary with limited effects on outcome [17,18].

The pre-operative angiogram, especially DSA, may provide information relative to the flow dynamics and how the aneurysm is being filled. If the ACoA aneurysm is being filled via the left A2, it can be technically difficult to visualize the neck from a right-sided approach and careful attention is needed about subarachnoid dissection from the right. It can be critical in these circumstances to ensure maximal bony removal of the basal skull base to optimize visualization.

Right-sided approach to ACoA aneurysm

There is an ongoing debate regarding the optimal side for approaching ACoA aneurysms during surgical intervention. Some experts argue that a right-sided approach is appropriate for all ACoA aneurysms since early proximal control over both A1s can be readily secured during the intradural journey. Exceptions to this approach may arise in unusual circumstances, such as a giant right-facing aneurysm with an isolated left A1 supply, and a known right brain dominance to avoid traversing in the region of the more eloquent hemisphere, or a left-sided intracerebral hemorrhage that dictates a specific approach to spare the only healthy basal frontal lobe. Sano et al. reported that factors for surgical approach to aneurysms include the predominance of A1, the direction of the A2 fork and the aneurysm, aneurysm size, and multiplicity. However, with proper patient positioning, EVD placement to reduce brain edema, and adequate drilling of the sphenoid and orbit, a right-sided approach over the non-dominant frontal lobe can be utilized irrespective of these factors [19].

In cases of giant aneurysms, the approach should be from the direction that enables early arrival at the aneurysm neck. Approaching from the side of dominant A1 is generally recommended, but for anteriorly projecting giant aneurysms, an interhemispheric approach from posterior to the aneurysm is typically preferred. Similarly, for high-positioned ACoA aneurysms with dome > 15 mm and neck > 10mm measured from the level of the anterior clinoid process, aneurysms with a diameter > 8 mm may be better accessed and clipped with less residual neck via an interhemispheric compared to a pterional approach [20].

Although postoperative hematomas and venous infarctions are rare after aneurysm surgery, they can cause severe disabilities including motor weakness, aphasia, alexia, and Gerstmann syndrome when the dominant hemisphere is affected depending on the region of injury [5]. As the left hemisphere is typically the dominant hemisphere, approaching unruptured ACoA aneurysms from the right side can be a viable option that minimizes the risk of functional disability [5]. Approaching aneurysms through the right hemisphere offers several benefits. Firstly, right-handed surgeons can reduce the risk of errors due to the optimization of surgical cones and the optimization of right-hand positioning during dissection and approach. Secondly, the potential for brain damage is minimized, as any retraction injuries or venous infarctions that may occur have a lower chance of resulting in clinical complications assuming the patient has a dominant left hemisphere [21]. Thirdly, wide exposure of both the aneurysm neck and bilateral A1 is possible from a right-sided approach, as surgeons can freely dissect the cisterns and H-complex without obstructing the operating field with their working hand. As a result, surgeons can apply the clip more thoroughly, without leaving any excess material around the aneurysm neck. In addition, complete identification and preservation of the hypothalamic branch is more feasible, reducing the risk of cognitive impairment [21,22].

In a retrospective study by Kim et al., 113 patients with unruptured ACoA aneurysms were treated using the pterional approach [5]. In this study, even though left A1 dominancy was found in 62.8% of patients, 81.4% of patients underwent craniotomy with a right pterional approach. The results showed that 92.9% of patients achieved an excellent outcome (Glasgow Outcome Scale (GOS) of 5). Complete clipping was achieved in 94.9% of patients in the right hemispheric approach group and in only 81.3% of patients in the left hemispheric approach group. Even in patients with left A1 dominancy, GOS outcomes did not differ between the approaching sides, and the rate of complete clipping was higher in the right-side approach group than in the left-side approach group. These findings suggested that the surgical results of the right-side approach are not inferior to those of the left-side approach, and the left-side approach for unruptured ACoA aneurysms may not be beneficial even in cases of left A1 dominancy. According to the authors, the reasons for this difference likely include not only the size of the aneurysm but also the wide exposure of both the aneurysm neck and the bilateral A1 in the right hemispheric approach [5]. Five cases in the right-side approach group and one case in the left-side approach group encountered cerebral contusion or venous infarction. Of those, only two patients experienced symptomatic complications such as contralateral hemiparesis or aphasia. The authors felt that utilizing the right hemispheric approach reduced the likelihood of venous infarction or retraction injury leading to clinical neurological deficits. If the left-side approach was chosen for most cases, the complication rate would have likely increased further due to increased retraction on the dominant hemisphere. Thus, we also recommend using a right-side approach when operating on an ACoA aneurysm.

Limitations

As a single-center case report and technical review, this report is limited. Further evaluation with prospective trials and evaluation outcome metrics comparing surgical approaches may be beneficial to elucidate differences in outcomes with alternative approaches. Similarly, there is intrinsic selection bias as this is a single institutional evaluation.

## Conclusions

ACoA aneurysms are the most common type of intracranial aneurysm, which pose a challenge to neurosurgeons due to their complex anatomy, variations, and proximity to important blood vessels and structures. Successful treatment of ACoA aneurysms requires careful consideration of the anatomical characteristics of the aneurysm and the adjacent blood vessels and perforating branches. Surgical clipping remains an important and effective treatment option. It is important to preserve perforators, as well as subcallosal and hypothalamic branches during surgery and to consider factors such as ACoA variation, A1 morphology, and the relationship between bilateral A1 and A2 in the formation of aneurysms. Preoperative planning considers the shape and size of the aneurysm, projection of the aneurysm, ruptured status, and the anatomical relationship with adjacent blood vessels. In our experience, with an understanding of nuances regarding patient positioning, exposure, methods of brain relaxation, and surgical equipment nearly all ACoA can be safely approached from the right-sided nondominant hemisphere.
